# Similarity measures-based graph co-contrastive learning for drug–disease association prediction

**DOI:** 10.1093/bioinformatics/btad357

**Published:** 2023-06-01

**Authors:** Zihao Gao, Huifang Ma, Xiaohui Zhang, Yike Wang, Zheyu Wu

**Affiliations:** College of Computer Science and Engineering, Northwest Normal University, No.967 Anning East Road, Lanzhou, 730070, China; College of Computer Science and Engineering, Northwest Normal University, No.967 Anning East Road, Lanzhou, 730070, China; Guangxi Key Laboratory of Trusted Software, Guilin University of Electronic Technology, No.1 Jinji Road, Guilin, 541004, China; College of Computer Science and Engineering, Northwest Normal University, No.967 Anning East Road, Lanzhou, 730070, China; College of Computer Science and Engineering, Northwest Normal University, No.967 Anning East Road, Lanzhou, 730070, China; College of Computer Science and Engineering, Northwest Normal University, No.967 Anning East Road, Lanzhou, 730070, China

## Abstract

**Motivation:**

An imperative step in drug discovery is the prediction of drug–disease associations (DDAs), which tries to uncover potential therapeutic possibilities for already validated drugs. It is costly and time-consuming to predict DDAs using wet experiments. Graph Neural Networks as an emerging technique have shown superior capacity of dealing with DDA prediction. However, existing Graph Neural Networks-based DDA prediction methods suffer from sparse supervised signals. As graph contrastive learning has shined in mitigating sparse supervised signals, we seek to leverage graph contrastive learning to enhance the prediction of DDAs. Unfortunately, most conventional graph contrastive learning-based models corrupt the raw data graph to augment data, which are unsuitable for DDA prediction. Meanwhile, these methods could not model the interactions between nodes effectively, thereby reducing the accuracy of association predictions.

**Results:**

A model is proposed to tap potential drug candidates for diseases, which is called Similarity Measures-based Graph Co-contrastive Learning (SMGCL). For learning embeddings from complicated network topologies, SMGCL includes three essential processes: (i) constructs three views based on similarities between drugs and diseases and DDA information; (ii) two graph encoders are performed over the three views, so as to model both local and global topologies simultaneously; and (iii) a graph co-contrastive learning method is introduced, which co-trains the representations of nodes to maximize the agreement between them, thus generating high-quality prediction results. Contrastive learning serves as an auxiliary task for improving DDA predictions. Evaluated by cross-validations, SMGCL achieves pleasing comprehensive performances. Further proof of the SMGCL’s practicality is provided by case study of Alzheimer’s disease.

**Availability and implementation:**

https://github.com/Jcmorz/SMGCL.

## 1 Introduction

Rapid advances in drug research and development over the past few decades, as well as public health emergencies, such as the outbreak of COVID-19, have forced researchers to explore effective ways to counter these risks. Computer-aided prediction of drug–disease associations (DDAs, a.k.a. drug repositioning) is becoming more appealing as it involves de-risked compounds, which could lead to lower total development expenses and shorter development schedules.

At present, the popular DDA prediction methods can be roughly divided into two categories: DDA prediction based on matrix decomposition and completion, and DDA prediction based on Graph Neural Networks (GNNs). For the methods based on matrix decomposition and completion, BNNR (Yang *et al.* 2019) integrates the drug–drug, drug–disease, and disease–disease networks and uses a bounded nuclear norm regularization method to complete the drug–disease matrix under the low-rank assumption; GRGMF (Zhang *et al.* 2020b) is an improved neural collaborative filtering framework, which learns the neighbor information for each node adaptively and draws support from existing external similarity information to enhance the prediction performance. For the methods based on GNNs, DRWBNCF (Meng *et al.* 2022) encodes known DDAs together with drug and disease neighborhood and neighbor interactions, allowing specific network features to be taken into account as well; MVGCN (Fu *et al.* 2022) constructs multiple views by combining different similarity networks with the biomedical bipartite network and uses a neighborhood information aggregation layer to aggregate the information of inter- and intra-domain neighbors in different views. Although the above methods have achieved promising performance, they all suffer sparsely labeled data problems due to the limited annotated data as wet experiments are expensive and time-wasting. These data are insufficient to induce accurate representations of drugs and diseases in most cases, leading to suboptimal performance.

A contrastive learning paradigm from the computer vision domain is one approach to addressing these difficulties (Wu *et al.* 2018, Chen *et al.* 2020), which aims to construct consistent and inconsistent view pairs via data augmentations, including cutout and color distortion (Howard 2014). Some researchers have made a preliminary attempt at graph data (Huang *et al.* 2021, Zhao *et al.* 2021). However, contrastive learning on drug repositioning has its unique challenges: (i) the graph of DDAs has fewer nodes and more sparse edges (a number of diseases might only be treated by one drug). Therefore, techniques with node/edge dropout are completely unavailable for DDA prediction. (ii) When creating self-supervision signals, most existing methods generally consider neighbors in isolation. We instead argue that interactions between neighboring nodes may reveal potential relations between them and the target node, and modeling such interactions can improve the target node representation to imply richer semantics.

To get over the aforementioned limitations, we enrich the DDA graph contrastive learning (GCL) by incorporating the drug–drug similarity graph and disease–disease similarity graph, motivated by the fact that the indications for similar drugs are often the same. On top of that, we propose an end-to-end Similarity Measures-based Graph Co-contrastive Learning (SMGCL) model for DDA prediction with three modules. The first module, “multi-source contrast views construction,” builds the known DDA view, the drug-similarity, and disease-similarity views (applying the nearest neighbors) by using three sources of data. The second module, “context-aware neighborhood aggregation,” uses a bilinear GNN to capture complicated local feature in the DDA view, and a global-aware attention mechanism to compensate for the receptive field issue in bilinear aggregation. The last module is “contrastive objective,” where we introduce a sampling mechanism to radically mine supervised signals for efficient co-contrastive learning. Furthermore, the prediction task and the contrastive learning task are unified under a “primary&auxiliary” learning paradigm. Cross-validation and extensive experiments on three benchmark datasets provide statistical evidence for the superiority of SMGCL over the baseline approaches, and further case study demonstrates the practicability of SMGCL.

## 2 Materials and methods

**Figure 1.**
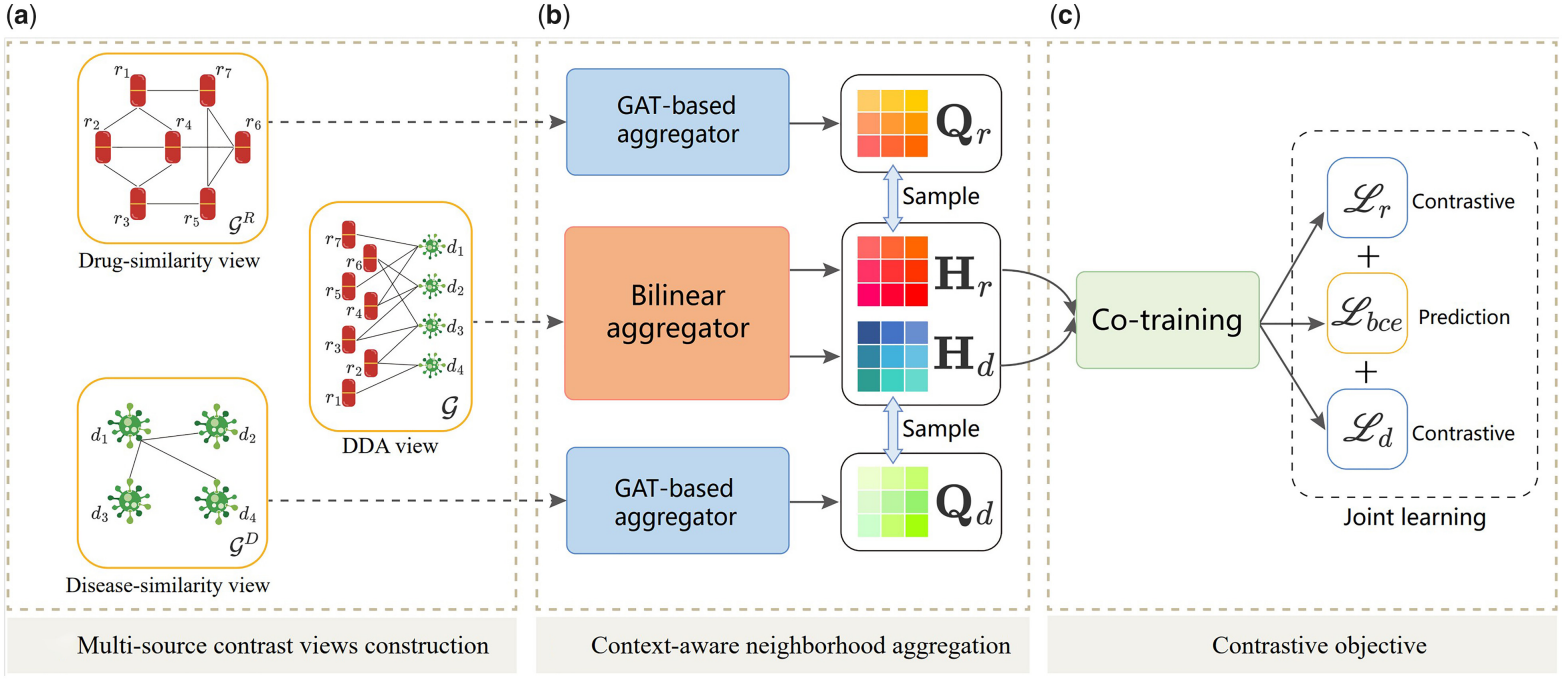
The framework overview of the proposed SMGCL. Solid rounded rectangles in (a) indicate three kinds of views, which are constructed from three different kinds of data. The DDA view is constructed on the known associations in the training set. Next, the node representation generated by the random walk with restart is transformed and applied as input to the model. Then, filled rounded rectangles in (b) indicate neural network encoders. For each type of node, we can get two kinds of representations by the different neural network encoders. Finally, we co-train the node representations, the prediction task and the contrastive learning task are unified under a primary&auxiliary learning paradigm in (c). Best viewed in color.

We denote vectors by lowercase boldface, matrices by uppercase boldface, and sets by uppercase calligraphic font. Thus, let $R=\{r_{1},r_{2},\ldots ,r_{N}\}$ denotes the set of drugs, where *N* is the number of drugs; $D=\{d_{1},d_{2},\ldots ,d_{M}\}$ denotes the set of diseases, where *M* is the number of diseases. The objective of DDA prediction is to learn a mapping function f((r,d)|ω):E→[0,1] from edges to scores, where ω is a parameter, in order to determine the probability that a given drug would be effective in treating a given disease. Figure 1 displays the architecture of the proposed method. Note that, the description on the whole model from the drug part, since the drug and disease parts are dual.

### 2.1 Multi-source contrast views construction

#### 2.1.1 DDA view

The DDA view can be regarded as an undirected graph G={V,E}, where V represents the set of nodes that correspond to drugs and diseases, E⊆V×V denotes the set of edges and indicates the existence of interaction between two kinds of nodes in V. Furthermore, the graph G can be represented as an incidence matrix $A\in {\left\{0,1\right\}}^{N\times M}$, where $A_{ij}=1$ if drug $r_{i}$ can treat disease $d_{j}$, otherwise $A_{ij}=0$.

#### 2.1.2 Similarity view

A tremendous deal of effort has gone into calculating the similarity of drugs or diseases. Taking the construction of drug-similarity view as an example, with the similarity of drugs, for a certain drug node $r_{i}$, we can select drugs with the top-*K* highest similarity as the neighbor nodes, which are the most similar to this drug in chemical structure, side effects, etc. In this way, the drug-similarity view is denoted as $G^{R}\in \{V^{R},E^{R}\}$ with *N* drugs, and its adjacency matrix $A^{R}\in {\left\{0,1\right\}}^{N\times N}$, where $A_{ij}^{R}=1$ if drug $r_{j}$ is the top-*K* nearest neighbor of drug $r_{i}$; otherwise $A_{ij}^{R}=0$. In the same way, the disease-similarity view is denoted as $G^{D}\in \{V^{D},E^{D}\}$ with *M* diseases, and its adjacency matrix $A^{D}\in {\left\{0,1\right\}}^{M\times M}$, where $A_{ij}^{D}=1$ if disease $d_{j}$ is the top-*K* nearest neighbor of disease $d_{i}$; otherwise $A_{ij}^{D}=0$. For descriptive purposes, we define terms that are used interchangeably throughout the literature: view is a synonym for graph.

### 2.2 Context-aware neighborhood aggregation

After views construction, we develop a context-aware neighborhood aggregation including two encoders, to capture both heterogeneous (homogeneous) and local (global) information. Each encoder is in charge of extracting useful information from one heterogeneous (homogeneous) graph to improve DDA prediction.

#### 2.2.1 Node feature extraction

Each column of the adjacency matrix of the similarity view can act as an initial feature vector for the corresponding node; however, these vectors may not capture the higher order connectivity information of the graph. For this reason, we run Random Walk with Restart (Tong *et al.* 2006) separately on drug-similarity matrix $A^{R}$ and disease-similarity matrix $A^{D}$ to enrich the initial embeddings for each node with local structure context. The process can be defined as the following recurrence equation: where α is the restart probability, $P^{R}$ is the probability transition matrix obtained from $A^{R}$ by column-wise normalization. $x_{r_{i}}^{\left(l\right)}$ is a column vector of drug node $r_{i}$, whose *i*th entry indicates the probability of reaching node *i* after *l* steps. $x_{r_{i}}^{\left(0\right)}\in R^{N}$ is a one-hot vector with dimensions of *N* where *i*th entry is 1 and 0 otherwise, which denotes the initial vector representation of drug $r_{i}$.


(1) $$x_{r_{i}}^{\left(l+1\right)}=(1-\alpha )P^{R}x_{r_{i}}^{\left(l\right)}+\alpha \cdot x_{r_{i}}^{\left(0\right)},$$

After approaching the steady-state, a single-layer perceptron is applied to obtain $e_{r_{i}}=\text{MLP}(x_{r_{i}}^{\infty })$ on $A^{R}$ for drugs, where $e_{r_{i}}\in R^{t}$ denotes the updated drug node representation with *t* dimensions and MLP contains single hidden layer. In the same way, we can obtain the disease node representation $e_{d_{j}}\in R^{t}$.

#### 2.2.2 DDA view encoder

GCN (Kipf and Welling 2017) assumes that neighboring nodes are independent of each other and utilizes the weighted sum to learn low-dimensional representations of nodes. We formulate a GA aggregator for target node *v* (drug *r* or disease *d*) as: where GA(⋅) is the non-linear aggregator, $\hat{N}(v)=\{v\}\cup \{i|A_{vi}=1\}$ denotes the extended neighbors of node *v*, which contains the node *v* itself. σ is a non-linear activation function. $a_{vi}$ is the weight of neighbor *i* and is defined as $\frac{1}{\sqrt{{\hat{d}}_{v}{\hat{d}}_{i}}}$, where ${\hat{d}}_{v}=|\hat{N}(v)|$ and ${\hat{d}}_{i}=|\hat{N}(i)|$. $W_{g}$ is the weight matrix to do feature transformation.


(2) $$h_{v}^{\left(GA\right)}=\text{GA}({\left\{e_{i}\right\}}_{i\in \hat{N}(v)})=\sigma \left(\sum_{i\in \hat{N}(v)}a_{vi}W_{g}e_{i}\right),$$

In addition, the co-occurrence of two neighboring nodes can be regarded as an important feature of the target node. However, the common GCNs ignore the possible interactions between neighboring nodes. Even if it is a Graph Attention Networks that can adaptively aggregate the information of neighboring nodes of different importance, it cannot extract the possible interaction features between neighboring nodes. At the same time, multiplying two vectors can effectively model the interactions by emphasizing the consistent information and weakening the divergent information (Zhu *et al.* 2020). Thus, we define a BA aggregator for target node *v* as: where BA(⋅) is the non-linear aggregator, $b_{v}=\frac{1}{2}{\hat{d}}_{v}({\hat{d}}_{v}-1)$ denotes the number of interactions for the node *v*, eliminating the bias of node degree to some extent with the normalization process. ⊙ is element-wise product and $W_{b}$ is the weight matrix to do feature transformation.


(3) $$\begin{array}{c} h_{v}^{\left(BA\right)}=\text{BA}({\left\{h_{i}\right\}}_{i\in \hat{N}(v)})\\ =\sigma \;\left(\frac{1}{b_{v}}\sum_{i\in \hat{N}(v)}\sum_{j\in \hat{N}(v)\&i<j}W_{b}e_{i}⊙W_{b}e_{j}\right), \end{array}$$

Then, the encoder which is built on the DDA view for message passing between drugs and diseases extracts indirect interactions in the local structure. Specifically, for target node *v*, the DDA view encoder is defined as: where β is a hyper-parameter to trade-off the strengths of the GA aggregator and BA aggregator.


(4) $$h_{v}=\beta \times h_{v}^{\left(GA\right)}+(1-\beta )\times h_{v}^{\left(BA\right)},$$

#### 2.2.3 Similarity view encoder

Previous drug repositioning research assumed that similar drugs would treat the same disease, but we argue that dissimilar drugs might also treat the same disease. To fully exploit this potential correlation, we design a global-aware strategy based on an attention architecture, which increases significant signals and weakens noisy signals when calculating the attention coefficient $\delta_{vi}$, to obtain node representations considering various perspectives. Specifically, the following two aspects are taken into account by the attention mechanism.

Firstly, we calculate the average representation of all nodes’ embedding in the similarity view. In order to explore the potential of drug treatment for non-indications, the node representation and average information representation are used to calculate the following attention score: where ${\text{att}}_{1}$ is a single-layer feedforward neural network with the LeakyReLU as activation function, $W_{1}$ is a transformation matrix, $\bar{e}$ represents the average node information by average pooling.


(5) $$\epsilon_{i}={\text{att}}_{1}(W_{1}e_{i}⊙\bar{e}),$$

Apart from the above, we extend the message passing process by the attention mechanism. If the drug neighbor node is more correlated with the target drug node, its contribution in aggregation toward the target node will be more significant and vice versa. where $W_{2}$ is a transformation matrix, ‖ denotes the concatenation operation, $e_{j}$ is the neighbor node representation of the node *v*, and ${\text{att}}_{2}$ is a single-layer feedforward neural network applying the LeakyReLU nonlinearity.


(6) $$\zeta_{ij}={\text{att}}_{2}(W_{2}e_{i}||W_{2}e_{j}),$$

Then, both the global and local score of each node is added following the additive attention mechanism (Bahdanau *et al.* 2015). Besides, softmax function is utilized to normalize coefficients across all choices of *j*, so as to make coefficients are able to directly compared between all nodes. The attention coefficients $\delta_{ij}$ between node *i* and node *j* can be calculated as: where $\epsilon_{j}$ determines the amount of information flow from *j* while $\zeta_{ij}$ decides the information target node *i* may receive. In this way, we can get another representation of drugs and diseases obtained on “drug-similarity view” and “disease-similarity view,” respectively, which are denoted as $q_{v}(v\in \{r,d\})$. The calculation is defined as: where $W_{3}$ is the weight matrix.

Remark: We elaborately describe the drug representation learning process here. Because the disease representation learning is a dual process, we omit it for brevity.

(7) $$\delta_{ij}=\mathrm{softma}x_{i}\left(\epsilon_{j}+\zeta_{ij}\right)=\frac{\;\text{exp}\;\left(\epsilon_{j}+\zeta_{ij}\right)}{\sum_{k\in N(i)}\;\text{exp}\;\left(\epsilon_{k}+\zeta_{ik}\right)},$$

(8) $$q_{i}=\sigma (\sum_{j\in N(i)}\delta_{ij}W_{3}h_{j}),$$

### 2.3 Generating prediction and model optimization

To reconstruct the associations between drugs and diseases, our decoder $f(e_{r_{i}},e_{d_{j}})$ is formulated as follows: where ${\hat{y}}_{r_{i},d_{j}}$ is the predicted probability score.


(9) $${\hat{y}}_{r_{i},d_{j}}=\text{MLP}(e_{r_{i}}⊙e_{d_{j}},h_{r_{i}},h_{d_{j}}),$$

DDA graph possesses two characteristics: (i) sparse edges (there is only a small number of existing DDAs) and (ii) limited nodes (the number of drugs and diseases are far less than those of users and items in the recommender systems). In order to make full use of all these information, we thus take all unknown drug–disease pairs as negative instances in the training set of each fold. Since there is no negative sampling, the setting of negative samples in the training set and the test set are the same. Furthermore, some of the existing studies (Zhao *et al.* 2021) sample the same number of unknown DDAs as that of the known association in the training set in some studies. We argue that the sampling strategy tends to adopt random sampling, which is likely to introduce unnecessary noise. Given that there are far fewer known DDAs than there are unknown or unseen DDAs, and since known DDAs have undergone extensive laboratory and clinical validation, they are highly reliable and crucial for enhancing predictive performance. Hence, our proposed SMGCL learns parameters by minimizing the weighted binary cross-entropy loss as follows: where (i,j) indicates the pair of drug $r_{i}$ and disease $d_{j}$, $S_{rd}^{+}$ denotes the set of all known DDAs, and $S_{rd}^{-}$ represents the set of all unknown or unseen DDAs. The balance factor $\eta =\frac{\left|S_{rd}^{-}\right|}{\left|S_{rd}^{+}\right|}$ emphasizes the importance of observed associations to mitigate the damage of data imbalance, where $\left|S_{rd}^{-}\right|$ and $\left|S_{rd}^{+}\right|$ are the number of pairs in $S_{rd}^{-}$ and $S_{rd}^{+}$. Moreover, instead of minimizing the weighted binary cross-entropy loss, we also consider the variant of our model, named SMGCL-NS, which minimizes the binary cross-entropy loss. It also means that the same number of unknown DDAs as known associations is sampled.


(10) $$\begin{array}{c} L_{\mathrm{bce}}=-\frac{1}{N\times M}(\eta \times \sum_{(i,j)\in S_{rd}^{+}}\;\text{log}\;{\hat{y}}_{r_{i},d_{j}}\\ +\sum_{(i,j)\in S_{rd}^{-}}(1-\text{log}\;{\hat{y}}_{r_{i},d_{j}})), \end{array}$$

### 2.4 Contrastive objective

#### 2.4.1 Mining self-supervision signals

Through the above section, we have constructed two view encoders over three views, each of which can deliver complimentary semantics to the other. As a result, it makes sense to improve each encoder by using the data from the other view. In this section, we illustrate how SMGCL enhances DDA prediction by mining valuable self-supervision signals. This can be accomplished by following the co-training architecture. Given a drug $r_{i}$ and disease $d_{j}$ in the DDA view, we choose their positive and negative drug samples within the same minibatch using its representation learned over the similarity view: where $s\mathrm{cor}e_{r}\in R^{M}$ denotes the predicted probability of each disease being cured to the drug *r* in the similarity view.


(11) $$\mathrm{scor}e_{r}=\mathrm{softmax}(Q_{d}q_{r}),$$

A natural intuition is that we may select highly confident diseases via calculated probabilities, i.e. top-*K* ranking diseases, so as to supervise the drug embedding in the similarity view as augmented ground truths. The positive sample selection is defined as: where $P_{d}^{K}$ denotes picking the corresponding diseases *d*, which are according to the top-*K* probability scores with the highest confidence.


(12) $$S_{r_{i}}^{d^{+}}=P_{d}^{K}\left(\mathrm{scor}e_{r_{i}}\right),$$

When it comes to picking negative samples, a simple intuition is to choose the diseases with the lowest scores. Nevertheless, this approach contributes minimally to the representation update and cannot distinguish and tailor complex and difficult samples. Thus, *K* negative samples are randomly chosen from diseases ranked in top 50% in $s\mathrm{cor}e_{r_{i}}$ excluding the positives to construct $S_{r}^{d^{-}}$. We argue that these diseases should be considered as hard negatives, suggesting finer information with slight possibility of false negatives that may deceive learning. Finally, the information samples used for disease embeddings are selected in the same way to get $S_{d_{i}}^{r^{+}}$ and $S_{d_{i}}^{r^{-}}$.

The positive and negative pseudo-labels for each drug and disease in the similarity view are repeatedly generated for every training batch. More hard negative samples are anticipated to be produced by repeating this procedure. Note that the encoders can evolve under the guidance of informative samples, recursively extracting more hard samples.

#### 2.4.2 Co-contrastive learning

With the generated pseudo-labels, the graph co-contrastive learning task for evolving the encoder can be performed by contrastive objects. We utilize NT-Xent (You *et al.* 2020) as our objective function to maximize the mutual information between the two views. Formally, the training objective for drug $h_{r_{i}}$ is as follows: where τ denotes the temperature parameter and sim(u,v) is the cosine similarity. In the same way, the training objective for disease $h_{d_{i}}$ is defined as:


(13) $$L_{r_{i}}=-\text{log}\;\frac{\sum_{d_{j}\in S_{r_{i}}^{d^{+}}}\left(e^{\mathrm{sim}\left(\left(h_{r_{i}},h_{d_{j}}\right)\right)/\tau }\right)}{\sum_{d_{k}\in S_{r_{i}}^{d^{+}}\cup S_{r_{i}}^{d^{-}}}\left(e^{\mathrm{sim}\left(h_{r_{i}},h_{d_{k}}\right)/\tau }\right)},$$

(14) $$L_{d_{i}}=-\text{log}\;\frac{\sum_{r_{j}\in S_{d_{i}}^{r^{+}}}\left(e^{\mathrm{sim}\left(\left(h_{d_{i}},h_{r_{j}}\right)\right)/\tau }\right)}{\sum_{r_{k}\in S_{d_{i}}^{r^{+}}\cup S_{d_{i}}^{r^{-}}}\left(e^{\mathrm{sim}\left(h_{d_{i}},h_{r_{k}}\right)/\tau }\right)}.$$

Finally, we unify the prediction task with the auxiliary SSL task. The total loss L is defined as: where λ is hyper-parameter to control the scale of the graph co-training.


(15) $$L=L_{\mathrm{bce}}+\lambda \cdot (L_{r}+L_{d}),$$

The weights are initialized in accordance with Glorot and Bengio (2010), and the model is optimized using the Adam optimizer (Kingma and Ba 2015). We train the model in a denoising setup by randomly dropping out edges with a fixed probability, which enables us to effectively generalize to the unseen data and avoid the model from over-fitting. For the graph convolution layers, we also use regular dropout.

## 3 Experiments

### 3.1 Experimental settings

#### 3.1.1 Datasets

We evaluate our model on three benchmark datasets: “Fdataset” (Gottlieb *et al.* 2011), “Cdataset” (Luo *et al.* 2016), and “LRSSL” (Liang *et al.* 2017), which are often used in DDA prediction. The basic statistics of the three datasets are shown in Table 1. Sparse ratio is defined as the ratio of the number of known associations to the number of all possible associations. Details of these benchmarks are in the Supplementary Material.

**Table 1.** Statistical details of the benchmark datasets.

Dataset	Number of drugs	Number of diseases	Number of associations	Sparse ratio
Fdataset	593	313	1933	0.0104
Cdataset	663	409	2352	0.0087
LRSSL	763	681	3051	0.0058

#### 3.1.2 Baseline methods

To evaluate the effectiveness of our proposed SMGCL, we compare it with various baseline methods: (i) matrix factorization and completion models including SCMFDD (Zhang *et al.* 2018), BNNR (Yang *et al.* 2019), DRIMC (Zhang *et al.* 2020a), and GRGMF (Zhang *et al.* 2020b); (ii) deep learning-based models including NIMCGCN (Li *et al.* 2020), LAGCN (Yu *et al.* 2021), DRWBNCF (Meng *et al.* 2022), and MVGCN (Fu *et al.* 2022). Details of these baseline methods are in the Supplementary Material.

#### 3.1.3 Evaluation metrics and parameters settings

To assess SMGCL’s overall performance, we adopt the Area Under the Receiver Operating Characteristic curve (AUROC) and the Area Under the Precision–Recall curve (AUPR) as primary metrics. It is meaningful to measure the characteristics of ROC and PR while treating the unknowns as true negatives since the actual associations are limited in comparison to the total number of unknowns. Details of each metric are in the Supplementary Material.

Our proposed SMGCL model uses the Adam optimizer. The values of all hyper-parameters refer to the practices of previous researchers and are finally determined by grid search, where the learning rate is set as 0.001, batch size is set as 64, restart probability *α* = .1, temperature τ=0.1, and scale control hyper-parameter λ=0.1. For trade-off hyper-parameter β, SMGCL has different optimal parameters for different benchmark datasets. On Fdataset, β=0.6; on Cdataset and LRSSL, β=0.8. Besides, all methods have been compared under the same evaluation settings. For the baseline models available for code disclosure, we run the code with reference to the best parameters reported in the original paper, and our results are consistent with those in publications. For the baseline models with unavailable codes, we report the results directly since we use the same datasets.

### 3.2 Overall performance

Following Li *et al.* (2020) and Zhang *et al.* (2020a), we adopt 10-fold cross-validation (10-CV) to evaluate the performance of prediction methods. In particular, for each 10-CV repetition, we calculate all evaluation metrics, and the final evaluation results are obtained by calculating the average evaluation metrics over 10 repetitions. The prediction model is constructed on the known associations in the training set and is used to predict the associations in the remaining fold as the test set. Besides, we deploy a *t*-test under AUROC and AUPR metrics. Table 2 reports the performance comparison results and statistical significance, in which SMGCL-NS means that the same number of unknown DDAs as known associations is sampled. We have the following observations:

**Table 2.** The average metrics of compared methods obtained in 10-CV.

Dataset	Fdataset		Cdataset		LRSSL	
	AUROC	AUPR	AUROC	AUPR	AUROC	AUPR
SCMFDD	0.7748	0.0510	0.7921	0.0514	0.7783	0.0358
BNNR	0.9298	0.4372	0.9338	0.4702	0.9267	0.3152
DRIMC	0.9091	0.3096	0.9333	0.3894	0.9314	0.2661
GRGMF	0.8047	0.5503			0.8157	0.4396
NIMCGCN	0.8281	0.3385	0.8508	0.4326	0.8294	0.2670
LAGCN	0.8586	0.1188	0.9144	0.1849	0.9336	0.1109
MVGCN	0.8527	**0.5582**	0.8617	**0.6302**	0.8493	**0.4431**
DRWBNCF	0.9245	0.4845	0.9404	0.5589	**0.9345**	0.3416
SMGCL	**0.9352** *	0.5486*	**0.9468** *	0.6256 *	0.9262*	0.3904*
SMGCL-NS	0.9284*	0.5244*	0.9369*	0.5816*	0.9136*	0.4374*

* Indicates *P*-value <.05 in the significance test. The best results are in bold, and the suboptimal results are underlined.

1. On three datasets, BNNR and DRIMC outperform expectations in terms of performance. Such performance might be attributed to a smaller number of nodes in DDA data compared to e-commerce and social recommendation data, which allows for the promising performance of BNNR and DRIMC on AUROC. In addition, as an improved neural collaborative filtering framework, GRGMF introduces two graph regularization terms to deal with nodes without any known link information, thus enhancing the learning of latent representations. This may greatly alleviate the influence of unbalanced data on the model and achieve suboptimal performance on AUPR. However, GRGMF does not explicitly model the connectivity in the embedding learning process, which easily leads to its poor performance on AUROC.
2. Compared with NIMCGCN and LAGCN, the performance of DRWBNCF verifies that modeling neighbor interactions can improve representation learning. MVGCN is the only model that uses contrastive learning apart from the proposed SMGCL. The difference with SMGCL is that MVGCN uses contrastive learning to obtain the initial representation of nodes, while SMGCL optimizes the contrastive objective and prediction task jointly. MVGCN obtains optimal performance on AUPR, which validates that contrastive learning can mitigate the impact of data imbalance. Surprisingly, in some cases, the performance of NIMCGCN, LAGCN, and MVGCN is worse than that of BNNR and DRIMC. The reason might be that NIMCGCN ignores the interaction of nodes in heterogeneous networks, and LAGCN indiscriminately mixes the network topology information of different domains (i.e. drug and disease domains), and MVGCN does not select the nearest neighbor of each node to construct the similarity view, which introduces a lot of noise information.
3. The AUROC obtained by SMGCL on Fdataset and Cdataset shows the best performance, on LRSSL shows great performance. Compared with GRGMF and MVGCN, the average AUROC of SMGCL increased by 15.54% and 9.55%, respectively. Moreover, in the context of imbalance classification, AUPR is also an indispensable evaluation metric. Compared with BNNR and DRWBNCF, the average AUPR of SMGCL increased by 27.96% and 12.98%, respectively. To clarify the advantages of SMGCL, more detailed comparison between SMGCL and MVGCN in Supplementary Section S2.4. Benchmarking comparison results show that SMGCL improves the comprehensive prediction performance thanks to combining the information of the known DDA is co-trained with the neighborhood and neighborhood interaction information of drugs and diseases under the framework of contrastive learning.

### 3.3 Model ablation

To evaluate the rationality of design sub-modules in our SMGCL framework, we consider three model variants as follows:

1. SMGCL without DDA view encoder (w/o-DE): We only use the similarity views to model drugs and diseases, removing the graph co-contrastive learning.
2. SMGCL without similarity view encoder (w/o-AE): We only use the DDA view to model drugs and diseases, removing the similarity views, interaction-aware similarity views, and the graph co-contrastive learning.
3. SMGCL without co-contrastive learning task (w/o-CL): We remove the graph co-contrastive learning task and only use simple summing of drug/disease embeddings on two views to get the final embedding.

**Figure 2.**
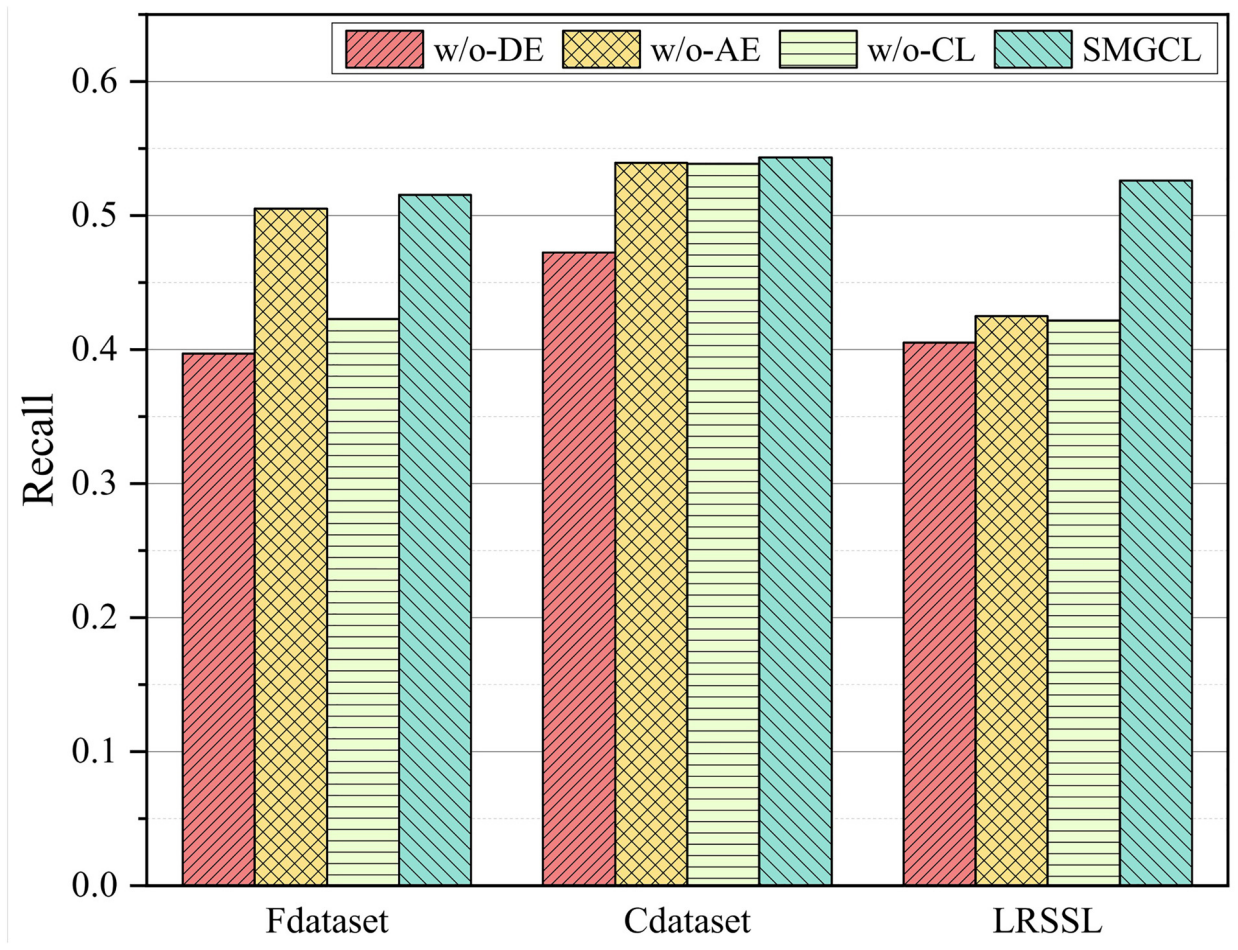
The Recalls of all compared approaches obtained in 10-CV.

As can be observed in Fig. 2, each component contributes to the final performance. The DDA view encoder contributes the most. When only using the DDA view encoder, the model achieves a suboptimal performance, which is much higher than the performance of the SMGCL without co-contrastive learning task on both the three datasets. This can demonstrate the effectiveness of modeling the interaction between neighbor nodes. By comparison, only using the similarity view encoder would lead to a huge performance degradation on three datasets. Surprisingly, removing the co-contrastive learning task and using the sum of drug/disease embeddings on two views to obtain the final embedding do not achieve suboptimal performance. This proves that contrastive learning can automatically mine labels, so as to maximize agreement between nodes in different view. According to this ablation study, we can conclude that a successful DDA prediction model should consider not only the interaction between drugs and diseases, but also the relationship between drugs and drugs, diseases and diseases.

### 3.4 Case study: approved drugs for Alzheimer’s disease determined by calculation

We conduct a case study for the neurodegenerative disease Alzheimer’s disease (AD), for which there are currently no effective treatments, in order to further evaluate the predictive capability of SMGCL. All of the known DDAs in the Fdataset are used as the training set and the unknown DDAs are used as the candidate set when trying to find possible AD drugs. Once the SMGCL predicts the probability of interaction of a given disease with all drug candidates, we rank the candidates according to that predicted probability, so that the top-ranked drug is the most likely to treat the disease.

We focus on the top 15 potential candidates for AD predicted by SMGCL in Table 3. For each drug, we show the DrugBank ID, canonical name and literature-reported evidence, which check the predicted DDAs. Then, we select three drugs in Table 3 to describe them in detail. Amantadine has antiviral, anti-Parkinson’s, and anti-pain activities. By promoting dopamine release from striatal dopaminergic nerve terminals and preventing its pre-synaptic reuptake, it has anti-Parkinsonian actions. Furthermore, Erkulwater and Pillai (1989) have proved that the mental status of two AD patients has obviously improved after treatment with amantadine. Haloperidol is a highly effective first-generation antipsychotic drug and one of the most commonly used antipsychotics in clinical practice today. Devanand *et al.* (1998) have conducted an experiment on the efficacy and side effects of haloperidol and placebo in the treatment of psychosis and disruptive behavior in patients with AD. Ultimately, the results have shown that haloperidol at a dose of 2–3 mg/day had a good therapeutic effect. Carbidopa is the levorotatory isomer of a synthetic hydrazine derivative of the neurotransmitter dopamine. Meyer *et al.* (1977) have performed serial clinical assessments and neuropsychological measures of functioning in 10 patients with severe dementia consisting of AD or multi-infarct dementia (MID) or both, who have taken Carbidopa. The results have demonstrated that one patient with AD + MID demonstrated clinical and psychological improvement.

**Table 3.** The top 15 SMGCL-predicted candidate drugs for AD.

Rank	Drug name	DrugBank IDs	Evidence
1	Amantadine	DB00915	Erkulwater and Pillai (1989)
2	Ropinirole	DB00268	Shaughnessy (2006)
3	Haloperidol	DB00502	Devanand *et al.* (1998)
4	Isoprenaline	DB01064	Ohm *et al.* (1991)
5	Carbidopa	DB00190	Meyer *et al.* (1977)
6	Risperidone	DB00734	Mintzer *et al.* (2006)
7	Scopolamine	DB00747	San Tang (2019)
8	Phenobarbital	DB01174	Brodie and Kwan (2012)
9	Dopamine	DB00988	Louzada *et al.* (2004)
10	Phenytoin	DB00252	Dhikav (2006)
11	Benzatropine	DB00245	NA
12	Pramipexole	DB00413	Bennett *et al.* (2016)
13	Terabenazine	DB04844	Kilbourn *et al.* (1993)
14	Carbamazepine	DB00564	Olin *et al.* (2001)
15	Ceftriaxone	DB01212	Zumkehr *et al.* (2015)

The underline indicates that we have made a detailed analysis and introduction of these drugs in the following.

Overall, a variety of evidence from clinical trials and other literature data have validated 14 of the top 15 predicted drugs (93% success rate), ordered by confidence scores.

## 4 Conclusion

In this study, we look into the potential of GCL to address the shortcomings of the traditional DDA prediction. In particular, an end-to-end SMGCL model is suggested to tap candidate drugs for diseases. To be specific, we learn the representation of drugs and diseases on three relevant views and then introduce a co-contrastive learning strategy that can sample positive samples and dig hard negative samples to generate accurate node representations. Finally, experiments on three benchmark datasets justify the advantages of our proposal regarding DDA prediction. The reliability of the newly discovered DDAs has been supported by case study.

Since the task of DDA prediction is closely related to biological safety and human health. It is crucial to design a reasonable negative sampling strategy for constructing a robust DDA prediction model. In future work, we will consider developing a proper negative sampling strategy for the DDA prediction task and analyze the performance improvement of the negative sampling strategy on different SOTA models.

## Supplementary Material

btad357_Supplementary_Data
