# Supplementary material for: Similarity measures-based graph co-contrastive learning for drug–disease association prediction
Source: Bioinformatics. 2023 Jun 1;39(6):btad357. doi: 10.1093/bioinformatics/btad357 (PMC10275904; doi:10.1093/bioinformatics/btad357)
Supplement: btad357_Supplementary_Data [file btad357_supplementary_data.zip › SMGCL_supplementary.pdf]

Supplementary Material

1 Implementation details of the experiments

1.1 Details of benchmark datasets

SMGCL has been evaluated on three benchmark datasets: *Fdataset* [1], *Cdataset* [2] and *LRSSL* [3], which are often used in DDA prediction.

- *Fdataset* [1] contains 1933 associations between 593 drugs taken from DrugBank [4] and 313 diseases listed in the OMIM database [5]. Moreover, the drug-disease data set includes two disease-similarity measures and five drug-similarity measures. Five drug similarity measures are implemented based on information about drug chemical structure, drug side effects, and drug target information. Based on the semantic similarity of disease phenotypes, the pairwise disease-similarity is calculated.
- *Cdataset* [2] contains 663 drugs registered in DrugBank, 409 diseases listed in OMIM database and 2352 known DDAs. Drug similarity is measured based on chemical structures and disease similarity is measured based on phenotypes using MimMiner [6].
- *LRSSL* [3] contains 763 drugs registered in DrugBank and 681 diseases from Nation Drug File-Reference Terminology (NDF-RT), which is part of the Unified Medical Language System (UMLS) [7]. Furthermore, it contains 3051 known DDAs. Drug similarity is measured based on chemical fingerprints defined in the PubChem database [8]. Disease similarity is measured based on the semantic similarity of disease phenotypes [9].

1.2 Details of baseline methods

SMGCL has been compared with various baseline methods: (1) Matrix factorization and completion models including SCMFDD [10], BNNR [11], DRIMC [12], GRGMF [13]; (2) Deep learning-based models including NIMCGCN [14], LAGCN [15], DRWBNCF [16], MVGCN [17].

- **SCMFDD** [10] provides similarity constraints to smooth the features after projecting DDAs into two low-rank spaces to identify latent features for drugs and diseases.

- **BNNR** [11] completes the DDA matrix under the low-rank supposition, builds a heterogeneous DDA network that combines the drug-drug, disease-disease, and DDA networks, and incorporates a regularization term to balance the approximation error and the rank properties.
- **DRIMC** [12] is an effective drug repositioning model based on the completion of the Bayesian induction matrix.
- **GRGMF** [13] is an improved neural collaborative filtering framework. It formulates a generalized matrix factorization model to make use of the latent patterns behind the observed links.
- **NIMCGCN** [14] uses GCNs to learn node embeddings of miRNA and disease. The learned embeddings are then applied in a neural inductive matrix completion model to rebuild an association matrix. Its initial application is to identify connections between miRNA and diseases. It later turned out to have enormous potential for drug repositioning.
- **LAGCN** [15] is a layered attention graph convolutional network, which is used for the DDA prediction.
- **MVGCN** [17] constructs multiple views by combining different similarity networks with the biomedical bipartite network and uses a neighborhood information aggregation layer to aggregate the information of inter- and intra-domain neighbors in different views.
- **DRWBNCF** [16] proposes a weighted bilinear graph convolutional network to encode known DDAs as well as neighborhood and neighbor interactions for drugs and diseases.

1.3 Details of evaluation metrics

Definition 1 (True Positive Rate (TPR)) Based on the confusion matrix, it represents the probability that samples which are true positive are predicted as positive samples. TP indicates that instances which are predicted as positive and are actually positive. FN indicates that instances which are predicted as negative but are actually positive.

$$TPR = \frac{TP}{TP + FN}, \tag{S1}$$

Definition 2 (False Positive Rate (FPR)) Based on the confusion matrix, it represents the probability that samples which are true negative is wrongly predicted as a positive sample. FP indicates that instances which are predicted as positive but are actually negative. TN indicates that instances which are predicted as negative and are actually negative.

$$FPR = \frac{FP}{FP + TN}, \tag{S2}$$

Definition 3 (Precision) Based on the confusion matrix, it represents the probability of being true positive samples among all the samples predicted to be positive.

$$Precision = \frac{TP}{TP + FP}, \tag{S3}$$

Definition 4 (Recall) Based on the confusion matrix, it represents the probability that samples which are true positive are predicted as positive samples. It is easy to find that TPR is equal to Recall.

$$Recall = \frac{TP}{TP + FN}, \tag{S4}$$

Definition 5 (Area Under Receiver Operating Characteristic (AUROC)) By calculating the TPR and FPR of the model under different thresholds, and taking the TPR as the vertical axis and the FPR as the horizontal axis, the ROC curve can be obtained. AUROC is the area surrounded by ROC curve, x axis and x=1. The larger the area, the better the performance.

Definition 6 (Area Under Precision-Recall (AUPR)) By calculating the precision and recall of the model under different thresholds, the PR curve can be obtained with precision as the vertical axis and recall as the horizontal axis. AUPR is the area enclosed by the PR curve and the coordinate axis. The larger the area, the better the performance.

2 More Experimental Results

2.1 Hyperparameter Study

To validate the effect of different parameter settings, we conduct experiments on three benchmark datasets to evaluate the performance of our proposed SMGCL framework with different configurations of key hyperparameters, i.e. the hidden state dimensionality t, the number K of the nearest neighbors, the trade-off hyper-parameter β.

2.1.1 Impact of Hidden State Dimensionality.

The hidden state dimensionality t is searched in the range of {8, 16, 32, 64, 128, 256}. Supplementary Figure S1 plots the AUPRs obtained by SMGCL with respect to different dimensions t. We can observe that as the embedding dimension increases from 8 to 64, the prediction performance improves due to the stronger representation feature space. However, a larger embedding dimensionality causes the performance of the SMGCL to decline and then climb slowly. This is caused by the model overfitting.

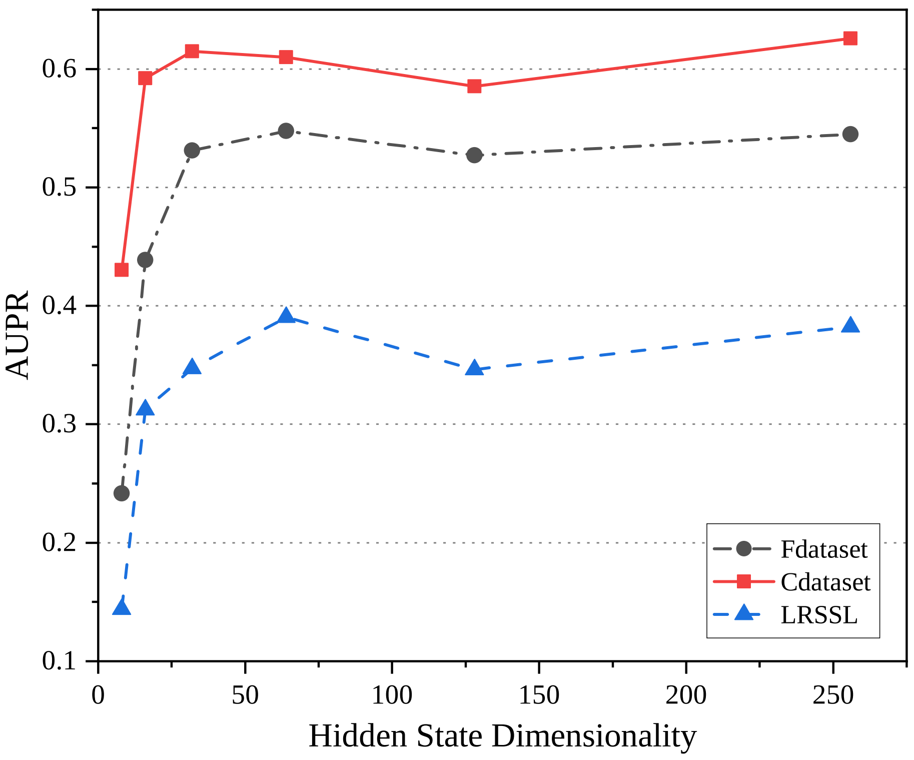

Figure S1: Impact of hidden state dimensionality.

2.1.2 Impact of the Number of the Nearest Neighbors.

The number K of the nearest neighbors is searched amongst $\{0, 2, 4, 6, 8, 10, 12, 14\}$. Supplementary Figure S2 plots the AUPRs obtained by SMGCL with respect to different numbers K of the nearest neighbors. We can observe that SMGCL achieves best AUPRs via setting K as 4, for three benchmark datasets, respectively. On the three benchmark datasets (i.e. Fdataset, Cdataset and LRSSL), when $K = 0$ (i.e. without neighbors information), the AUPRs obtained by SMGCL are 0.4509, 0.5447 and 0.3283, respectively. Furthermore, we verify that when the graph is a fully connected graph (i.e. K is set as the number of all neighbors), these values are 0.0583, 0.0393 and 0.0283. These findings explain how nearest neighbors can accurately predict the probability of a link between a specific drug and disease.

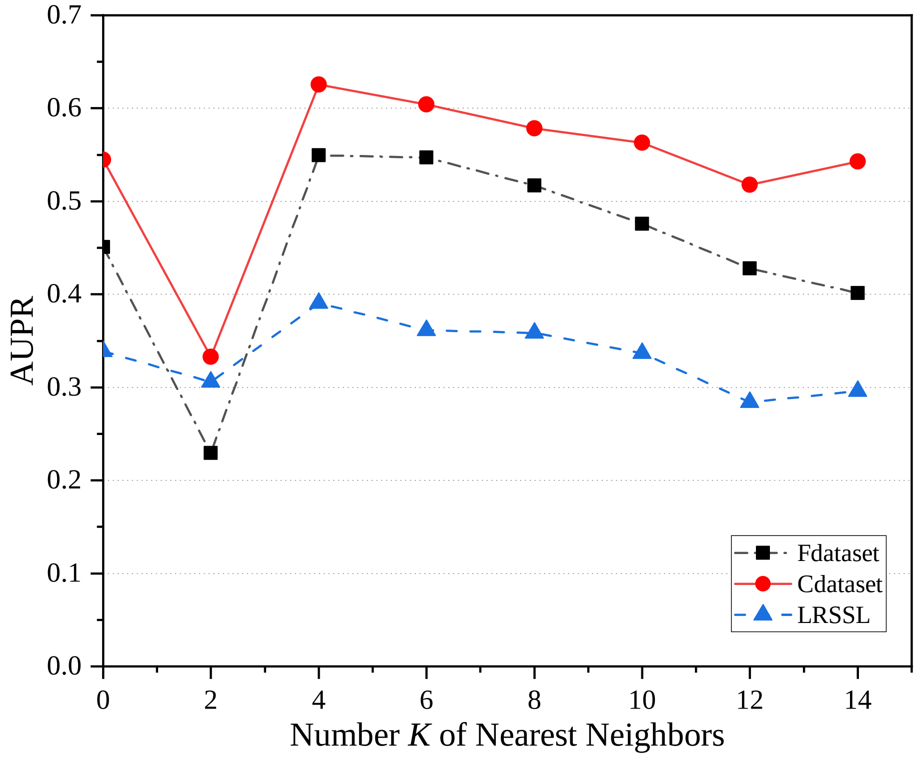

Figure S2: Impact of the number of the nearest neighbors.

2.1.3 Impact of the trade-off hyper-parameter β.

The trade-off hyper-parameter β is searched in the range of $\{0, 0.2, 0.4, 0.6, 0.8, 1\}$. Supplementary Figure S3 plots the AUPRs obtained by SMGCL with respect to different β values. Note that smaller β means more contributions from the BA; DDA view encoder will downgrade to vanilla GCN with only the GA aggregator by setting $\beta = 1$, while being fully dependent on the BA by setting $\beta = 0$. We have the following observations: (1) DDA view encoder outperforms vanilla GCN in most cases. It again verifies that the BA is capable of capturing the complex patterns of information propagation, which are hard to reveal by the linear aggregator individually. (2) Surprisingly, the performance of DDA view encoder with is worse than the performance when is set to the optimal value. One possible reason is that the BA mainly serves as the complementary component to the linear aggregator, hardly working alone to achieve the comparable performance.

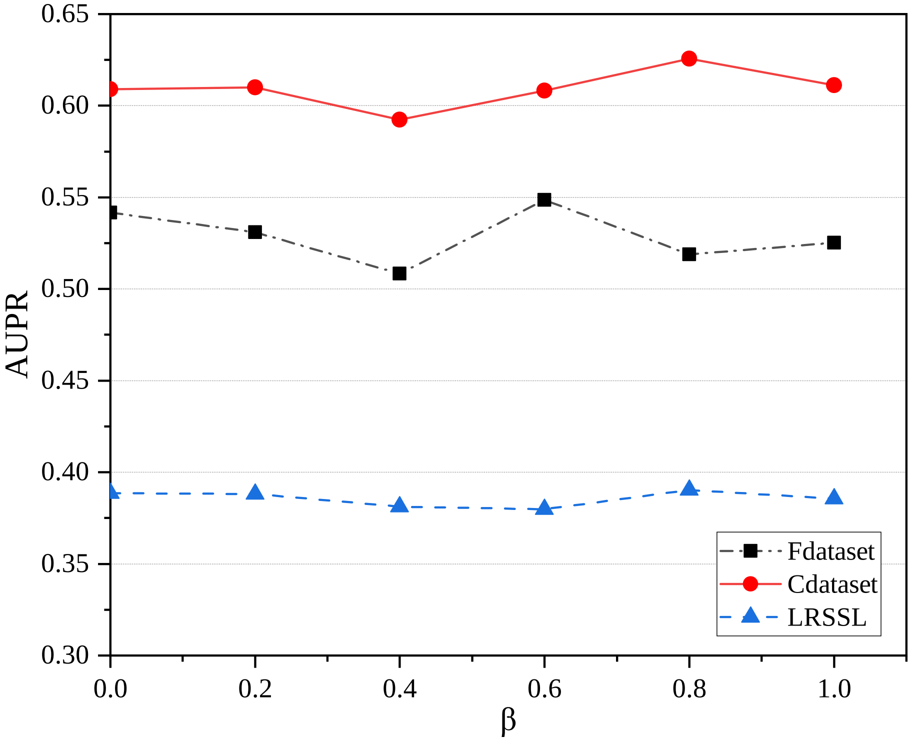

Figure S3: Impact of the trade-off hyper-parameter β.

2.2 Ablation Study

This section aims to answer the following question. **Q1:** *Compared with GATs* [18], *what are the advantages of BA aggregator?* We have replaced the BA aggregator with GATs, which we call "SMGCL-GAT". **Q2:** *Is ϵ in Eq. (7) indispensable?* We have compared the models with and withot ϵ.

Table S1: The AUROCs and AUPRs of SMGCL, SMGCL-GAT and w/o-ϵ obtained in 10-fold CV. * indicates p-value < 0.05 in the significance test.

**Datast**	**Fdataset**		**Cdataset**		**LRSSL**	
	AUROC	AUPR	AUROC	AUPR	AUROC	AUPR
**SMGCL-GAT**	0.9228*	0.5321*	0.9410*	0.6161*	0.9223*	0.3778*
**w/o-ϵ**	0.9304*	0.5375*	0.9425*	0.6100*	0.9133*	0.3623*

A1: From Table S1 (**Q1**), we can observe that GAT is capable of deciding the amount of information transmitted by neighboring nodes to the target node by

considering the similarity score between the target node and neighboring nodes. Still, it cannot extract the possible interaction features between neighboring nodes, which can be regarded as an essential attribute of the target node. On the contrary, the BA aggregator can effectively capture the interactions between neighboring nodes and deliver the captured information to the target node.

A2: Results in Table S1 (**Q2**) show that using simple local attention aggregation without considering the global information may ignore the potential of non-indication drug treatment. Therefore, the superiority of SMGCL is not merely from effectively sensing the information of local neighbor sand ϵ is empirically indispensable.

2.3 Comparison with MVGCN

2.3.1 Metrics Anaysis

It needs to be admitted that AUPR is indeed a very important metric in the context of imbalance classification. This is because, for the PR curve and ROC curve, the calculation of recall and TPR is the same, but the calculation of precision and FPR is quite different. Specifically, given a small number of positive samples and large number of negative samples, when the model identifies more negative samples as positive samples, the precision of the model will drop greatly, thus the PR curve will have a low Y-intercept, resulting in a low AUPR value. At the same time, the FPR of the model increases slightly, suggesting that AUPR is often more sensitive than AUROC.

Then, we analyze the following two situations, that is, *When will the model have a high AUROC value and a low AUPR value?* and *When will the model have a low AUROC value and a high AUPR value?*

a. *When will the model have a high AUROC value and a low AUPR value?*

   Considering a binary classification problem, the distribution of classes in the dataset is highly unbalanced. If a model is designed to classify all examples as negative, it will achieve a high TNR and a low FPR, resulting in a high AUROC score. However, such a model would have very low recall and precision for the positive class, resulting in a low AUPR score.

b. *When will the model have a low AUROC value and a high AUPR value?*

   Considering a binary classification problem, the distribution of classes in the dataset is highly unbalanced. A model that is designed to predict positive class more often may have a higher recall and precision for the positive class, leading to a high AUPR score. However, this may sacrifice the cost with a higher FPR, leading to a lower AUROC score.

Jointly analyzing the performances of MVGCN and SMGCL in Table 2, we have the following four findings:

1. MVGCN is more inclined to predict a sample as a positive sample than SMGCL;

2. The number of true positive samples predicted by MVGCN is more than that predicted by SMGCL, *i.e.*, $TP_{MVGCN} > TP_{SMGCL}$;

3. For all the samples predicted to be positive, the percentage of samples predicted correctly by MVGCN is higher than that by SMGCL, *i.e.*, $Precision_{MVGCN} > Precision_{SMGCL}$;

4. The number of negative samples of MVGCN prediction error is higher than that of SMGCL, and the difference between the negative samples that are incorrectly predicted by the two models is much larger than the difference between the positive samples that they predict correctly, *i.e.*, $FP_{MVGCN} > FP_{SMGCL}, FP_{MVGCN} - FP_{SMGCL} \gg TP_{MVGCN} - TP_{SMGCL}$;

Indeed, drug-disease association prediction can be applied in many practical scenarios, such as drug discovery, assisting physicians in prescribing in clinical practice, and patient self-service access to drug recommendation services from online healthcare service platforms. By treating more unrelated drug-disease pairs as related, MVGCN shows its advantage for drug discovery applications, since it can indeed provide more drug candidates for the next wet trials. However, SMGCL is more suitable for both the assisted physician prescribing and patient self-service access in terms of drug recommendation services from online health service platforms. No matter how high the precision value is, as long as the number of FPs is high, this is unacceptable for any patient. We believe that the improvement of the precision value should not be preconditioned by an increase in the number of FPs.

2.3.2 Model Comparison

Firstly, we briefly introduce the process of MVGCN framework:

1. Because a biological entity may have multiple similarity networks, MVGCN combines similarity networks with biomedical bipartite networks to construct multi-view heterogeneous networks, and then perform DGI on the two networks to obtain node attributes as initial embedding;

2. The neighborhood information aggregation (NIA) layer is proposed. Specifically, for a certain view, the representation of the l-level node is obtained by the weighted summation of the inter- and the intra-domain representation of the l-level node. The inter-domain representation is calculated on bipartite networks, and the intra-domain representation is calculated on the similarity network. MVGCN has designed two methods to get the node representation on the view, one is to get the node representation on all layers by average pooling, and the other is to directly use the node representation on the last layer;

3. Finally, the final node embedding is obtained by integrating multiple views, and then it is fed to the discriminator to predict the existence of links.

We select three datasets from the original paper of MVGCN as shown in Table S2, each of which can construct a different number of views with close sparsity. Then, we report the performance of these from the original paper of MVGCN as shown in Table S3.

Table S2: The statistics of three datasets (The content is derived from the original paper of MVGCN).

**Dataset**	**Type**	**Dimension**	**Links**	**Sparsity (%)**	**P**	**Q**
LiangDDA	DDA	763×681	3051	99.41	3	1
LuoDTI	DTI	708×1512	1923	99.82	4	3
LiuDTI	DTI	542×504	463	99.83	1	1

Table S3: AUC scores of prediction models evaluated on the independent test sets (The content is derived from the original paper of MVGCN).

**Model**	**LiangDDA**	**LuoDTI**	**LiuDTI**
MVGCN-II	0.8299	0.8908	0.7129
MVGCN-avg	0.8380	0.8933	0.7096
MVGCN	0.8518	0.9030	0.7141

According to the process of MVGCN framework, it is clear that for LiangDDA, MVGCN can build $3 \times 1 = 3$ views; For LuoDTI, MVGCN can build $4 \times 3 = 12$ views; For LiuDTI, MVGCN can construct $1 \times 1 = 1$ view. Jointly analyzing the above two tables, we can conclude that the performance of the model on the same sparse dataset depends largely on the number of views that can be built. Once similarity networks are not available to describe biological entities, the performance of MVGCN will be greatly reduced. In contrast, our SMGCL is less dependent on similarity networks. As can be seen from Table 2, when sufficient data cannot be obtained, our model still maintains consistent superior performance, which once again proves the contribution of our model in solving the problem of data sparsity.

In order to compare SMGCL with MVGCN more fairly, we modify the way of obtaining the initial representation of nodes from the similarity network by random walk to the way of MVGCN, that is, implementing self-supervised learning strategy from bipartite networks. We have named this variant SMGCL-DGI, and deploy t-test under AUROC and AUPR indicators. Table S4 shows the results of SMGCL-DGI on AUROC and AUPR, and the statistical significance of our model against MVGCN.

As shown in the Table S4, we are surprised to find that the performance of SMGCL-DGI on AUPR is equivalent to that of MVGCN, especially in Fdataset. In addition, the performance of SMGCL-DGI on AUROC is further improved

Table S4: The average metrics of SMGCL-DGI obtained in 10-CV. * indicates p-value < 0.01 in the significance test.

**Datast**	**Fdataset**		**Cdataset**		**LRSSL**	
	AUROC	AUPR	AUROC	AUPR	AUROC	AUPR
**MVGCN**	0.8527	0.5582	0.8617	0.6302	0.8493	0.4431
**SMGCL**	0.9352*	0.5486*	0.9468*	0.6256*	0.9262*	0.3904*
**SMGCL-DGI**	0.9362*	0.5401*	0.9468*	0.6283*	0.9273*	0.4407*

than that of SMGCL. In the last sub-section, we have pointed out that AUPR is often more sensitive than AUROC, and the correct classification of a small number of samples may greatly improve the value of AUPR, but it is difficult to significantly improve the performance on AUROC. For the more sensitive AUPR index, SMGCL-DGI maintains the performance equivalent to that of MVGCN, and for AUROC, SMGCL and all variants have made great improvements compared with MVGCN. All in all, the proposed model can take into account both positive and negative samples and achieve satisfactory comprehensive performance.

Last but not least, it should be noted that when the model adopts self-supervised learning strategy to obtain the initial representation of nodes, the time consumption in training the model will also increase significantly. Therefore, the method of obtaining the initial representation of nodes still needs to be combined with specific practical application scenarios.

3 Discussion

Contrastive learning is first popularized in the field of computer vision. Researchers usually use cutout [19] and color distortion [20] to construct similar and dissimilar view pairs. When this technology is applied to graph data, the edge dropout strategy naturally becomes a way for researchers to construct contrastive auxiliary views. However, does the edge dropout on graph data have a positive or negative impact on contrastive learning? In this section, we test it through a specific experiment.

To prevent the unexplained experimental outcomes caused by random edge dropout, we design an interaction-aware edge dropout, based on the assumption that there is universal homogeneity in drug repositioning. That is, there are numerous correlations between drugs and diseases. Since similarity views are a reflection of homophily (e.g., drug-similarity graph is more likely to connect diseases that can be treated with the same drug, and vice versa), we use them to augment data in order to capture homophily for self-supervision.

Following this line, by aligning the drug-similarity view $\mathcal{G}^R$ and disease-similarity view $\mathcal{G}^D$ with DDA view $\mathcal{G}$, we can readily get two augmented graph $\mathcal{G}^R_{aug} \in \{\mathcal{V}^R, \mathcal{E}^R_{aug}\}$ and $\mathcal{G}^D_{aug} \in \{\mathcal{V}^D, \mathcal{E}^D_{aug}\}$. The edge of the former can be interpreted as a drug with a similar chemical structure that can treat the same disease, while the latter can be interpreted as a disease with similar semantic information that can be treated with one drug. The two aforementioned graphs can be effectively generated using matrix multiplication. Let $\mathbf{A}^R_{aug} \in \mathbb{R}^{N\times N}$ and $\mathbf{A}^D_{aug} \in \mathbb{R}^{M\times M}$ denote the adjacency matrices of the augmented graphs $\mathcal{G}^R_{aug}$ and $\mathcal{G}^D_{aug}$ respectively. Due to the augmented two views being coded in the same way, we call them separately *drug-auxiliary view* and *disease-auxiliary view* for the convenience. They can be calculated by:

$$\mathbf{A}^R_{aug} = (\mathbf{A}\mathbf{A}^\top) \odot \mathbf{A}^R, \quad \mathbf{A}^D_{aug} = (\mathbf{A}^\top\mathbf{A}) \odot \mathbf{A}^D, \tag{S5}$$

The multiplication $\mathbf{A}\mathbf{A}^\top$ ($\mathbf{A}^\top\mathbf{A}$) accumulates the paths connecting two drugs (diseases) via the same disease (drug). The Hadamard product $\odot$ guarantees that the relations in $\mathbf{A}^R_{aug}$ and $\mathbf{A}^D_{aug}$ are subsets of the relations in $\mathbf{A}^R$ and $\mathbf{A}^D$ respectively. Thus, Eq. (S1) can be viewed as a case of bootstrap sampling with the complementary information from $\mathbf{A}$.

Then, we replace the similarity views in SMGCL with the constructed auxiliary views, and learn the node representation on the DDA view, drug auxiliary veiw and disease auxiliary view, and implement co-contrastive learning. For convenience, we call this model SMGCL-ED. The final experimental results are shown in Table S2.

Table S5: The AUROCs and AUPRs of SMGCL-ED and SMGCL obtained in 10-fold CV.

**Method**	**Dataset**	**AUROC**	**AUPR**
**SMGCL-ED**	*Fdataset*	0.9226	0.4932
	Cdataset	0.9351	0.5106
	LRSSL	0.9108	0.2927
**SMGCL**	*Fdataset*	0.9352	0.5486
	Cdataset	0.9468	0.6256
	LRSSL	0.9262	0.3904

Jointly analyzing Table 2 and Table S5, we find that the performance of SMGCL-ED with edge dropout strategy on AUROC is slightly lower than that of the optimal baseline method. Except on Fdataset, the performance on AUPR is not as good as the optimal baseline method. In addition, compared with SMGCL, SMGCL-ED is generally inferior to SMGCL in three data sets, especially in AUPR. We believe that this result is due to the fact that constrastive learning can indeed alleviate the problem of label scarcity to a certain extent. However, because of sparse data, edge dropout will still greatly destroy the connectivity of graphs, and weaken the advantages of contrastive learning in solving label scarcity and mining supervision signals.

References

[1] Gottlieb, A., Stein, G.Y., Ruppin, E., Sharan, R.: Predict: a method for inferring novel drug indications with application to personalized medicine. Molecular systems biology **7**(1), 496 (2011)

[2] Luo, H., Wang, J., Li, M., Luo, J., Peng, X., Wu, F.X., Pan, Y.: Drug repositioning based on comprehensive similarity measures and bi-random walk algorithm. Bioinformatics **32**(17), 2664–2671 (2016)

[3] Liang, X., Zhang, P., Yan, L., Fu, Y., Peng, F., Qu, L., Shao, M., Chen, Y., Chen, Z.: Lrssl: predict and interpret drug–disease associations based on data integration using sparse subspace learning. Bioinformatics **33**(8), 1187–1196 (2017)

[4] Wishart, D.S., Feunang, Y.D., Guo, A.C., Lo, E.J., Marcu, A., Grant, J.R., Sajed, T., Johnson, D., Li, C., Sayeeda, Z., et al.: Drugbank 5.0: a major update to the drugbank database for 2018. Nucleic acids research **46**(D1), D1074–D1082 (2018)

[5] Hamosh, A., Scott, A.F., Amberger, J.S., Bocchini, C.A., McKusick, V.A.: Online mendelian inheritance in man (omim), a knowledgebase of human genes and genetic disorders. Nucleic acids research **33**, D514–D517 (2005)

[6] Van Driel, M.A., Bruggeman, J., Vriend, G., Brunner, H.G., Leunissen, J.A.: A text-mining analysis of the human phenome. European journal of human genetics **14**(5), 535–542 (2006)

[7] Bodenreider, O.: The unified medical language system (umls): integrating biomedical terminology. Nucleic acids research **32**, D267–D270 (2004)

[8] Wang, Y., Xiao, J., Suzek, T.O., Zhang, J., Wang, J., Bryant, S.H.: Pubchem: a public information system for analyzing bioactivities of small molecules. Nucleic acids research **37**, W623–W633 (2009)

[9] Wang, D., Wang, J., Lu, M., Song, F., Cui, Q.: Inferring the human microrna functional similarity and functional network based on microrna-associated diseases. Bioinformatics **26**(13), 1644–1650 (2010)

[10] Zhang, W., Yue, X., Lin, W., Wu, W., Liu, R., Huang, F., Liu, F.: Predicting drug-disease associations by using similarity constrained matrix factorization. BMC bioinformatics **19**(1), 1–12 (2018)

[11] Yang, M., Luo, H., Li, Y., Wang, J.: Drug repositioning based on bounded nuclear norm regularization. Bioinformatics **35**(14), i455–i463 (2019)

[12] Zhang, W., Xu, H., Li, X., Gao, Q., Wang, L.: Drimc: an improved drug repositioning approach using bayesian inductive matrix completion. Bioinformatics **36**(9), 2839–2847 (2020)

[13] Zhang, Z.C., Zhang, X.F., Wu, M., Ou-Yang, L., Zhao, X.M., Li, X.L.: A graph regularized generalized matrix factorization model for predicting links in biomedical bipartite networks. Bioinformatics **36**(11), 3474–3481 (2020)

[14] Li, J., Zhang, S., Liu, T., Ning, C., Zhang, Z., Zhou, W.: Neural inductive matrix completion with graph convolutional networks for mirna-disease association prediction. Bioinformatics **36**(8), 2538–2546 (2020)

[15] Yu, Z., Huang, F., Zhao, X., Xiao, W., Zhang, W.: Predicting drug–disease associations through layer attention graph convolutional network. Briefings in Bioinformatics **22**(4), bbaa243 (2021)

[16] Meng, Y., Lu, C., Jin, M., Xu, J., Zeng, X., Yang, J.: A weighted bilinear neural collaborative filtering approach for drug repositioning. Briefings in Bioinformatics **23**(2), bbab581 (2022)

[17] Fu, H., Huang, F., Liu, X., Qiu, Y., Zhang, W.: Mvgcn: data integration through multi-view graph convolutional network for predicting links in biomedical bipartite networks. Bioinformatics **38**(2), 426–434 (2022)

[18] Veličković, P., Cucurull, G., Casanova, A., Romero, A., Lio, P., Bengio, Y.: Graph attention networks. stat **1050**, 20 (2017)

[19] Devries, T., Taylor, G.W.: Improved regularization of convolutional neural networks with cutout. CoRR (2017), `http://arxiv.org/abs/1708.04552`

[20] Szegedy, C., Liu, W., Jia, Y., Sermanet, P., Reed, S.E., Anguelov, D., Erhan, D., Vanhoucke, V., Rabinovich, A.: Going deeper with convolutions. In: IEEE Conference on Computer Vision and Pattern Recognition, CVPR. pp. 1–9. IEEE Computer Society (2015)

Algorithm: SMGCL algorithm.

Input: DDA view $\mathcal{G}$; drug-similarity view $\mathcal{G}^R$; disease-similarity view $\mathcal{G}^D$; maximum training epochs T.

Output: Mapping function $f((r,d)|\omega) : \mathcal{E} \rightarrow [0,1]$ from edges to scores.

Calculate $\mathbf{e}_v$ via Eq.(1)
$t \leftarrow 1$
repeat
    for $v \in \mathcal{V}$ **do**
        Calculate $\mathbf{h}_v$ via Eq.(4)
    end for
    for $v \in \mathcal{V}$ **do**
        Calculate $\mathbf{q}_v$ via Eq.(8)
    end for
    Obtain the prediction matrix $\hat{\mathbf{Y}}$ via Eq.(9)
    Calculate $\mathcal{L}_{bce}$ via Eq.(10)
    for $r_i \in \mathcal{R}$ **do**
        Calculate $\mathcal{L}_{r_i}$ via Eq.(13)
    end for
    for $d_j \in \mathcal{D}$ **do**
        Calculate $\mathcal{L}_{d_j}$ via Eq.(14)
    end for
    Update ω by optimizing Eq.(15)
    $t \leftarrow t + 1$
until $t > T$ or Eq.(16) is converged
return $f((r,d)|\omega)$
