# Supplementary figures and images for: Similarity measures-based graph co-contrastive learning for drug–disease association prediction
Source: Bioinformatics. 2023 Jun 1;39(6):btad357. doi: 10.1093/bioinformatics/btad357 (PMC10275904; doi:10.1093/bioinformatics/btad357)

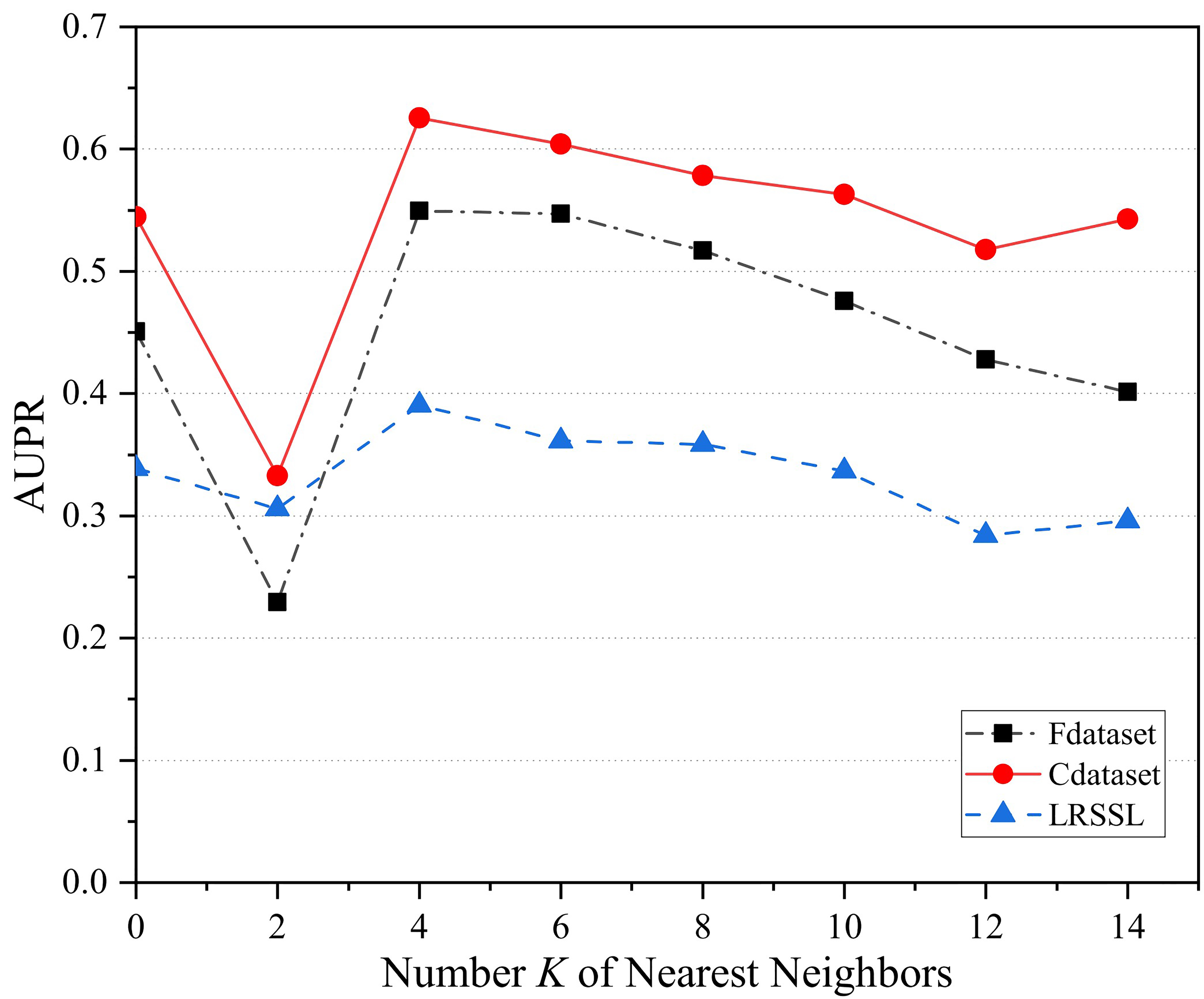

Supplement: btad357_Supplementary_Data [file btad357_supplementary_data.zip › Figure_s2.jpg]

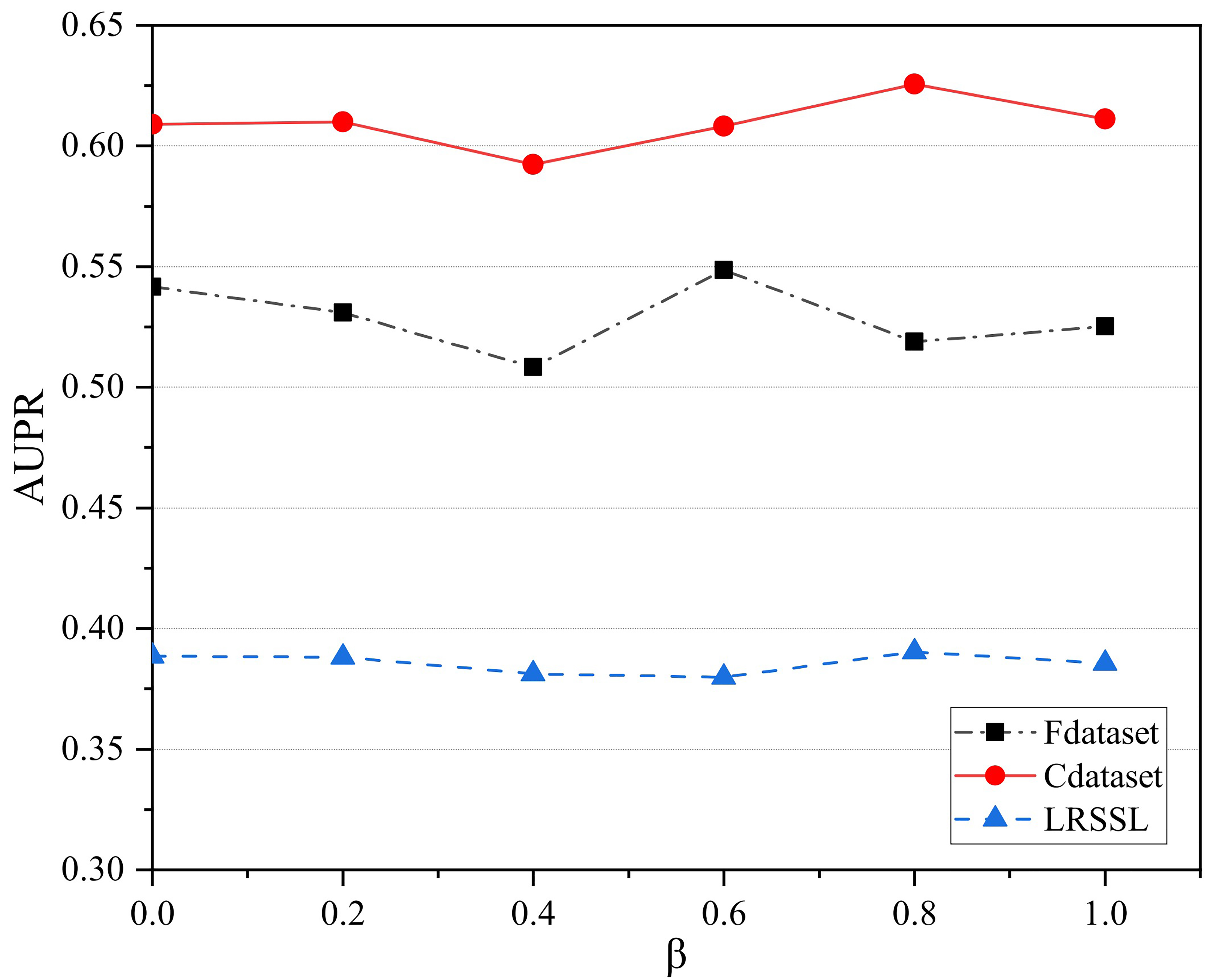

Supplement: btad357_Supplementary_Data [file btad357_supplementary_data.zip › Figure_s3.jpg]

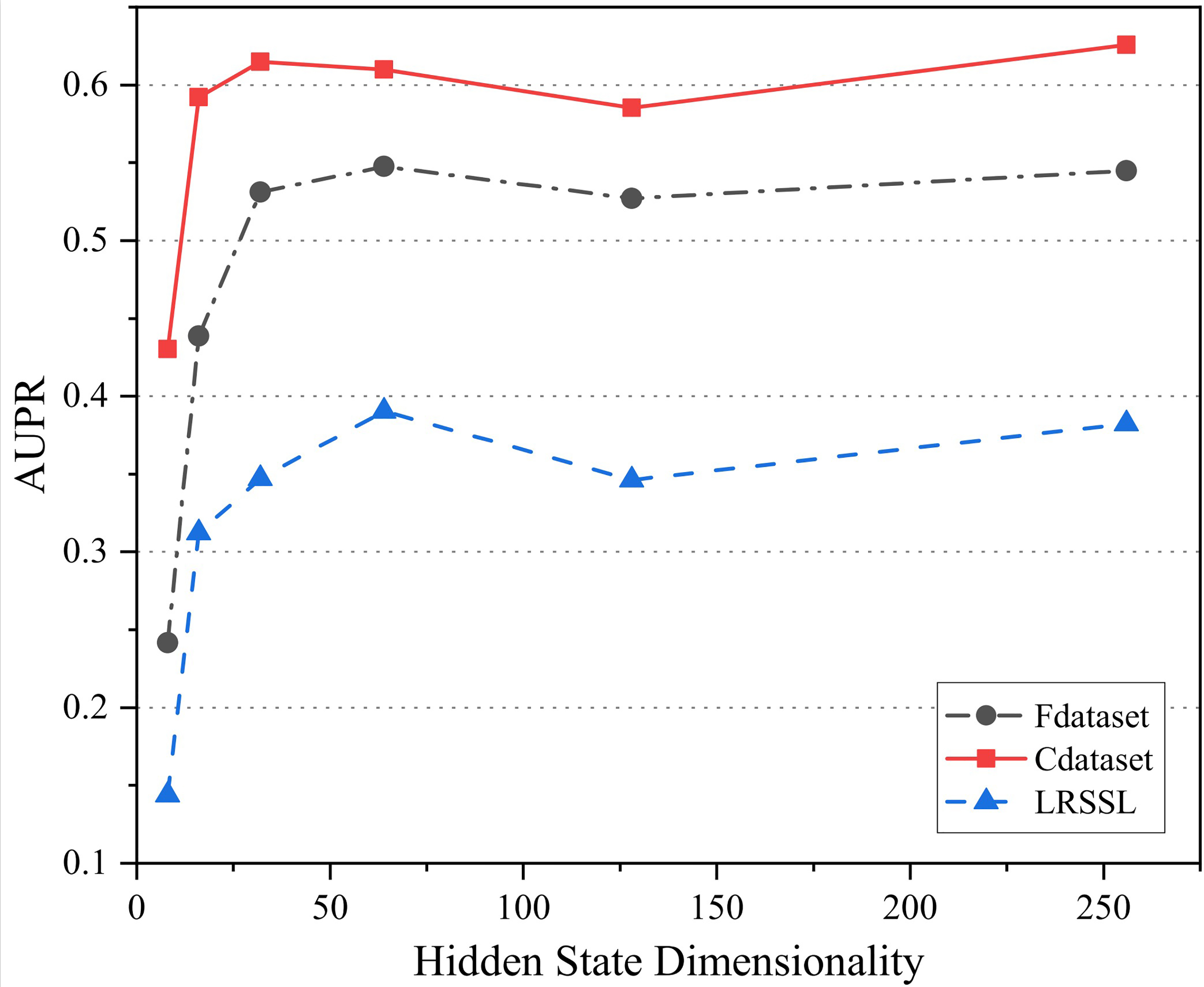

Supplement: btad357_Supplementary_Data [file btad357_supplementary_data.zip › Figure_s1.jpg]
